#  Actitudes de vecinos acerca del tratamiento y la vida en comunidad de personas con problemáticas severas de salud mental en Argentina 

**DOI:** 10.1590/0102-311XES083123

**Published:** 2023-10-13

**Authors:** Elena García, Guadalupe Ares-Lavalle, Mariana Borelli, Marina A. Fernández, Martín Agrest, Sara Ardila-Gómez

**Affiliations:** 1 Centro de Salud Mental Comunitaria E. Pichón Riviere, La Plata, Argentina.; 2 Facultad de Psicología, Universidad de Buenos Aires, Buenos Aires, Argentina.; 3 Hospital José A. Estéves, Ministerio de Salud, Temperley, Argentina.; 4 Proyecto Suma, Buenos Aires, Argentina.; 5 Consejo Nacional de Investigaciones Científicas y Técnicas, Buenos Aires, Argentina.

**Keywords:** Vivienda, Servicios Comunitarios de Salud Mental, Actitud, Inclusión Social, Housing, Community Mental Health Services, Attitude, Social Inclusion, Habitação, Serviços Comunitários de Saúde Mental, Atitude, Inclusão Social

## Abstract

La reforma psiquiátrica se encuentra en proceso en la región de América Latina y el Caribe. Específicamente en Argentina, el modelo de salud mental comunitaria está en construcción, siendo aún observable la presencia de internaciones psiquiátricas prolongadas, principalmente en hospitales neuropsiquiátricos. Resulta así necesario monitorear la reforma psiquiátrica, siendo una de las vías para ello el análisis de las actitudes de la sociedad hacia la internación psiquiátrica prolongada como modalidad de atención en salud mental. Así, se realizó un estudio observacional analítico en la Provincia de Buenos Aires, Argentina, en el año 2021, en donde se analizaron las actitudes de vecinos de personas que tuvieron internaciones psiquiátricas prolongadas y que recibían apoyos a la vivienda. Se hicieron cuestionarios a vecinos y no vecinos, indagándose por las actitudes hacia la internación psiquiátrica prolongada como modalidad de tratamiento, así como la distancia social hacia personas que tuvieron internaciones psiquiátricas, y también entrevistas a profundidad con informantes clave de barrios en donde habitan personas con problemáticas severas de salud mental y que reciben apoyos a la vivienda. No se identificaron diferencias estadísticamente significativas en las actitudes en relación a la internación psiquiátrica prolongada como modalidad de tratamiento de vecinos y no vecinos, ni tampoco respecto a la distancia social hacia personas que tuvieron internaciones psiquiátricas. Los informantes clave condicionaron su valoración sobre la internación prolongada, y valoraron el rol de los equipos de apoyo para posibilitar la vida en comunidad.

## Abstract (en)

A psychiatric reform is underway in Latin America and the Caribbean. Specifically in Argentina, a model of community mental health is being built, and prolonged psychiatric hospitalizations are still taking place, especially in neuropsychiatric hospitals. Therefore, it is necessary to closely monitor the psychiatric reform. One of the possible ways to monitor the reform is by analyzing society’s attitudes towards prolonged psychiatric hospitalization as a mean of mental health treatment. Thus, an analytical observational study was conducted at the Buenos Aires Province, Argentina, in 2021, to analyze the behavior of neighbors of people who had prolonged psychiatric hospitalizations and who received housing support. Questionnaires were applied to neighbors and non-neighbors, addressing the behaviors toward prolonged psychiatric hospitalization as a mean of treatment, social distance toward people who were hospitalized, as well as specific interviews with key informants from neighborhoods where people with severe mental health disorders and who receive housing support live. Based on the answers of neighbors and non-neighbors, no statistically significant differences were identified in behaviors toward prolonged psychiatric hospitalization as a mean of treatment, nor for social distance in relation to people who were hospitalized. Key informants conditioned their assessment of prolonged hospitalization and valued the role of support teams in making community life viable.

## Resumo

Uma reforma psiquiátrica está em curso na América Latina e Caribe. Especificamente na Argentina, um modelo de saúde mental comunitária está sendo construído, ainda acontecendo internações psiquiátricas prolongadas, principalmente em hospitais neuropsiquiátricos. Faz-se necessário, portanto, o acompanhamento da reforma psiquiátrica. Uma das formas de fazê-lo é analisando as atitudes da sociedade frente à internação psiquiátrica prolongada como forma de tratamento da saúde mental. Assim, foi realizado um estudo observacional analítico na Província de Buenos Aires, Argentina, no ano de 2021, onde foram analisadas as atitudes de vizinhos de pessoas que tiveram internações psiquiátricas prolongadas e que receberam apoio habitacional. Foram aplicados questionários a vizinhos e não vizinhos, questionando as atitudes em relação à internação psiquiátrica prolongada como forma de tratamento, bem como a distância social em relação às pessoas que foram internadas, e também entrevistas específicas com informantes-chave de bairros onde vivem pessoas com problemas graves de saúde mental e que recebem apoio habitacional. Com base nas respostas de vizinho e não vizinhos, não foram identificadas diferenças estatisticamente significantes nas atitudes em relação à internação psiquiátrica prolongada como forma de tratamento, nem em relação à distância social em relação às pessoas que foram internadas. Os informantes-chave condicionaram sua avaliação da hospitalização prolongada e valorizaram o papel das equipes de apoio na viabilização da vida comunitária.

## Introducción

En la región de América Latina y el Caribe, con ritmos, procesos y énfasis diferenciales, la reforma de la atención psiquiátrica tiene un largo recorrido, con desarrollos técnico-políticos y de implementación. Algunas experiencias de reforma fueron previas al hito de la Declaración de Caracas de 1990, pero a partir de ésta, adoptaron un envión significativo. A la par del desarrollo de instrumentos normativos, aumentó la conciencia por los derechos de las personas con problemáticas de salud mental, se reformaron hospitales psiquiátricos tradicionales, se fortaleció la internación en hospitales generales, y se desplegaron diferentes apoyos para que las personas con problemáticas de salud mental ejerzan sus derechos. Ello fue fortalecido por la Convención de los Derechos de las Personas con Discapacidad, asentada en el modelo de vida independiente y de los servicios de apoyo que buscan garantizar el derecho a elegir, posibilitar la vida comunitaria y evitar la institucionalización ^1^^,^^2^^,^^3^^,^^4^^,^^5^^,^^6^.

Con el cierre o transformación de los hospitales psiquiátricos tradicionales regional y mundialmente, es estimable que un número creciente de personas que antes hubiesen tenido como destino la internación prolongada o de por vida, hoy vivan en la comunidad. Ello se debe a que, o bien salieron de los hospitales psiquiátricos tradicionales o nunca fueron internados prolongadamente al contar con otra oferta de servicios ^7^. También, se debe considerar las barreras de acceso y la brecha de tratamiento en salud mental, la cual se estima que para trastornos mentales moderados a severos es del 74,7% en América Latina y el Caribe ^8^. Así, la experiencia creciente de la vida en comunidad de personas con problemáticas severas de salud mental ha tenido efectos en diferentes grupos: las mismas personas con problemáticas de salud mental, sus familiares, los servicios sociales y de salud y sus trabajadores y, en un sentido más amplio, la sociedad en su conjunto ^9^^,^^10^^,^^11^^,^^12^.

Este último grupo - la sociedad - resulta de interés al ser donde se vive, ejerce y posibilita de modo cotidiano la inclusión social de las personas con problemáticas de salud mental ^13^^,^^14^. Más allá del debate técnico o político respecto a la vida en comunidad de las personas con problemáticas de salud mental, es en la sociedad en donde se concreta y se materializa la experiencia de vivir en comunidad. En este sentido, se cuenta con estudios que analizan la evolución de las actitudes de la población general hacia el tratamiento en salud mental, a la par de los procesos de reforma psiquiátrica. Una revisión sistemática ^15^ encontraba que, con el avance de la reforma, la internación era más aceptada como opción de tratamiento para problemáticas consideradas graves, como la esquizofrenia. También hay estudios en donde se midió en diferentes momentos las actitudes y distancia social hacia personas con problemáticas severas de salud mental, en particular, esquizofrenia. Una revisión sistemática sobre las actitudes de la población general hacia la enfermedad mental no encontró cambios positivos en las actitudes entre 1990 y 2010 ^16^.

Asimismo, mediciones en 1996 y 2006 en población general de los Estados Unidos, no encontraron disminuciones significativas en ninguno de los indicadores de estigma empleados y, de hecho, encontraron un aumento en la reticencia a tener como vecino a una persona con esquizofrenia (de 34% a 45%) ^17^. Una medición similar en el año 2018 encontró que la reticencia a tener como vecino a una persona con esquizofrenia había aumentado al 49% y que si se preguntaba respecto a vivir cerca de residencias compartidas (“*group homes*”) la reticencia era del 33% para 1996 y del 36% para 2006 y 2018 ^18^. Por su parte, un estudio de Inglaterra sobre el efecto de una campaña anti-estigma realizó mediciones sobre distancia social entre los años 2009 y 2015 y encontró una disminución en la distancia social deseada en todas las dimensiones medidas, incluyendo el vivir cerca de una persona con problemas de salud mental ^19^. A su vez, diferentes estudios en población general en Argentina, encontraron reticencia a tener como vecina a una persona con enfermedad mental o esquizofrenia en el 47% de la población estudiada en 1964 ^20^, en el 13,5% en 2011 ^21^; y en el 40% en 2022 ^22^.

Los efectos de la reforma psiquiátrica en la sociedad pueden también analizarse empleando categorías intermedias, como el barrio o vecindario en donde habitan las personas con problemáticas de salud mental, pudiéndose estudiar a tal nivel las actitudes de los vecinos de las personas con problemáticas de salud mental. Los vecinos, además de la definición concreta por proximidad de quien vive “en la puerta del lado” o en la misma calle, son una de las fuentes posibles de apoyo social con la que cuentan las personas. Si bien el nivel de intimidad emocional en tales interacciones tiende a ser bajo, se producen con ellos interacciones frecuentes ^23^. Así, para los vecinos la vida en comunidad de personas con problemáticas de salud mental haría parte de su realidad cotidiana, por lo cual identificar sus actitudes en relación a las personas con problemáticas de salud mental y el tratamiento en salud mental es una vía posible para comprender los avances de la reforma psiquiátrica en una sociedad dada. El cortometraje “votamos” ^24^ retrata los ejes de las potenciales resistencias a la vecindad de una persona anónima, con antecedentes de internación psiquiátrica.

Los estudios, que han abordado las actitudes de los vecinos de viviendas con apoyo en donde pasaban a vivir personas con problemáticas de salud mental tras internaciones prolongadas, han descripto cambios en el tiempo, con resistencia inicial a la instalación de tales viviendas y disminución posterior ^25^. También, se ha descripto que la resistencia frente a la presencia de viviendas con apoyo era mayor entre personas que no contaban en su barrio con dichas viviendas que en la de aquellos en donde había tales residencias, siendo así mayor la oposición anticipada que la real ^26^^,^^27^. Un estudio en Argentina, en el cual se comparó a vecinos y no vecinos de mujeres que habían salido de un hospital psiquiátrico y vivían en residencias compartidas, encontró una asociación entre la aceptación hacia las personas con enfermedad mental y ser vecino de tales residencias ^28^. Por su parte, un análisis sobre las actitudes de residentes de vecindarios en donde se ubican servicios residenciales terapéuticos en Brasil, respecto a los pacientes psiquiátricos y los servicios de salud mental, encontró que las actitudes tendían a ser entre neutrales y positivas. Específicamente, el 98,2% no estuvo de acuerdo en que los “*enfermos mentales*” debían ser aislados de la comunidad, y un 94,6% estuvo de acuerdo en que “*nadie tiene derecho a excluir a los pacientes vecinos*” ^29^ (p. 270).

Es importante señalar que en la región de América Latina y el Caribe los procesos de reforma psiquiátrica están en etapas diferentes de consolidación de la salud mental comunitaria, en los diferentes países y también al interior de estos, por lo cual la pregunta por los efectos de la reforma psiquiátrica en la sociedad, aquí operacionalizada como comunidad vecinal, sigue teniendo vigencia. Por ello, resulta necesario monitorear periódicamente las actitudes frente a determinadas cuestiones o grupos poblacionales ya que las actitudes no son estáticas y sus cambios no necesariamente son lineales. Pueden relacionarse, por ejemplo, con el surgimiento de otros grupos como depositarios de discriminación social, con su lugar en la agenda de los medios de comunicación, o con las transformaciones en la experiencia directa, en el sentido de no solo imaginar o haber escuchado como es una personas con problemática severas de salud mental viviendo en la comunidad, sino de haber tenido la posibilidad de convivir comunitariamente con ella. Así, el objetivo de este estudio fue indagar las actitudes de vecinos de personas con problemáticas severas de salud mental sobre tales personas y su vida en comunidad, así como sobre la internación prolongada como modalidad de tratamiento en salud mental.

## Métodos

### Tipo de estudio

Se realizó un estudio observacional analítico. Para ello se comparó a un grupo de vecinos de personas con problemáticas severas de salud mental con un grupo de no vecinos de perosnas con problemáticas severas de salud mental. Las hipótesis fueron que los vecinos de personas con problemáticas severas de salud mental, en comparación con no vecinos, tienen: (1) actitudes más positivas respecto al tratamiento comunitario y la vida en comunidad de personas con problemáticas severas de salud mental; y (2) actitudes más negativas respecto a la internación psiquiátrica prolongada como forma de tratamiento en salud mental.

### Lugar del estudio

Se tomó como eje para el muestreo a tres programas que brindan apoyos a la vivienda a personas que tuvieron internaciones psiquiátricas prolongadas. Fueron criterios de muestreo la accesibilidad de información a tales programas, que tenían un tiempo de funcionamiento de más de 20 años al momento del estudio, y el ubicarse en la misma jurisdicción de nivel provincial, considerando el grado de autonomía de las provincias, al ser Argentina un país Federal. Tales programas, y las viviendas de las personas a quienes brindan apoyos, se localizan en centros urbanos medianos (menos de 1.000.000 de habitantes) de la Provincia de Buenos Aires. Dos de tales programas se ubican en municipios pertenecientes al Área Metropolitana de Buenos Aires, conglomerado urbano que concentra el 35% de la población del país; el tercero se ubica en la región sur de la Provincia de Buenos Aires. Cabe señalar que dos de los programas se vinculan a hospitales neuropsiquiátricos públicos, dando apoyo a las personas que son dadas de alta de dichos hospitales y que, generalmente, no cuentan con apoyo familiar o medios económicos para el alta. En uno de tales programas, la modalidad de vivienda es predominantemente grupal, y en el otro se cuenta con viviendas compartidas y con alquiler de habitaciones en pensiones. El tercer programa es coordinado por una organizaciones no gubernamentales (ONG) y se vincula a un servicio de internación en salud mental de un hospital general, siendo la modalidad de vivienda grupal.

Es importante señalar que, en Argentina, la reforma de la atención psiquiátrica se enmarca en la Ley Nacional de Salud Mental del año 2010, y que en la Provincia de Buenos Aires desde el año 2020 se viene implementando un programa de adecuación de los hospitales neuropsiquiátricos públicos a dicha normativa, enmarcado en un plan provincial ^30^. Ello ha implicado la externación de personas internadas prolongadamente. Según datos oficiales, a junio de 2022, 340 personas vivían en dispositivos residenciales comunitarios, vinculados a los hospitales en proceso de reforma ^31^.

### Participantes

En una primera etapa se realizaron entrevistas con trabajadores de los programas; en una segunda etapa, cuestionarios a vecinos y no vecinos de viviendas de personas que recibían apoyo por parte de estos programas; y, en una tercera etapa, entrevistas a comerciantes de los vecindarios en los cuales se ubican las viviendas que reciben apoyos. Los resultados de la etapa uno ya han sido publicados ^32^ y aquí se presentan los resultados de las etapas restantes.

Para la segunda etapa, se realizó para cada programa un mapeo de las viviendas en donde residían personas a las que se les brinda apoyos. Se trataban en su totalidad de viviendas compartidas en las que residía más de una persona que tuvo internación psiquiátrica prolongada, estando algunas de tales viviendas ubicadas en casas o departamentos grupales y otras en pensiones. El mapeo se realizó sobre un total de 16 viviendas. Se excluyeron viviendas localizadas en el predio de un centro comunitario en salud mental y viviendas unipersonales. La muestra calculada inicialmente fue de 128 cuestionarios: 64 a vecinos y 64 a no vecinos. Dicho cálculo se basó en la estimación de cuatro cuestionarios por vivienda que recibía apoyo, y un número equivalente de cuestionarios a no vecinos.

Se definió como vecino a una persona adulta que viviese en las dos viviendas de los costados o en las dos viviendas de enfrente de la residencia que recibía apoyos por parte de los programas. Si en los domicilios seleccionados no era posible realizar el cuestionario, se procedía a timbrar en el domicilio inmediatamente contiguo. La tolerancia máxima para realizar el cuestionario era la de la misma calle de la vivienda que recibía apoyos. Se excluyó a las edificaciones comerciales. En el caso de viviendas localizadas en edificios, se definió como vecinos a las personas residentes en los departamentos contiguos, siendo la tolerancia máxima la de residentes en el mismo edificio. A su vez, se estimó realizar un número equivalente de cuestionarios (n = 64) a “no vecinos”, definidos como personas que vivían en zonas socioeconómica y urbanísticamente similares a las de las viviendas con apoyos en las que residían personas com problemáticas severas de salud mental, con una distancia mínima de dos cuadras de las viviendas en donde habitaban personas que recibían apoyos.

De los 128 cuestionarios planificados, fue posible hacer 119 (93% de lo planificado), 55 de vecinos (86% de lo planificado) y 64 de no vecinos (el 100% planificado). Se registró una mayor dificultad para efectuar los cuestionarios a vecinos de viviendas ubicadas en edificios. Los cuestionarios se realizaron de modo simultáneo un sábado de agosto de 2021 en horas de la mañana. Fueron realizados por estudiantes de carreras vinculadas a la salud, quienes recibieron capacitación específica para esta tarea, y quienes no sabían si los domicilios asignados para realizar los cuestionarios correspondían a viviendas de vecinos o de no vecinos.

Para la etapa tres, se consideró como referentes barriales a comerciantes, ya que, por su trabajo, interactúan de manera permanente con los habitantes de los vecindarios, siendo observadores privilegiados de lo que en ellos ocurre. Para la selección de los comerciantes a entrevistar, en el cuestionario a los vecinos se preguntó por tres negocios del barrio a los que se solía acudir. Dicha información se analizó comparativamente, y después se verificó con los programas de apoyo si se trataba de comercios que también frecuentaban las personas con problemáticas severas de salud mental residentes en el barrio. Se realizaron siete entrevistas en comercios de vecindarios en donde se ubican viviendas de dos de las experiencias de apoyo, entre octubre y diciembre de 2021. En la tercera experiencia no se realizaron las entrevistas, pues los comercios a los que acudían las personas que recibían apoyos correspondían a grandes supermercados y no a pequeños negocios. Todos los comercios en donde se realizaron las entrevistas pertenecían al rubro de venta de alimentos: panaderías, verdulerías, almacenes y tiendas pequeñas (kioscos) y las entrevistas fueron realizadas por integrantes del equipo de investigación.

### Instrumentos

El cuestionario a vecinos y no vecinos constaba de 57 preguntas: datos sociodemográficos (preguntas 1 a 16); experiencia en el barrio y vínculos con los vecinos (preguntas 17 a 32); actitudes sobre personas con diversas problemáticas (preguntas 33 a 38); actitudes en relación a personas con internaciones psiquiátricas prolongadas, definiéndose como internación prolongada a aquella con una duración superior a seis meses (preguntas 39 a 52); referidas a la pandemia y sus efectos en la salud mental (preguntas 53 a 57). Las preguntas sobre la pandemia se agregaron a posteriori, pues el estudio se planificó pre-pandemia y el trabajo de campo se realizó durante la pandemia por COVID-19. Los resultados específicos sobre los efectos de la pandemia ya han sido publicados ^33^.

Las preguntas sobre vínculos con los vecinos fueron diseñadas con base en la *Escala de Cohesión Barrial de Buckner*^34^, realizándose modificaciones culturales a la misma. Se incluyeron 8 de las 19 preguntas del cuestionario original, correspondientes a las dimensiones de vecindad (interacciones entre los vecinos de una manera cooperativa) y de sentido psicológico de comunidad (sensación de pertenencia a una comunidad geográfica) de tal instrumento. Seis de las preguntas sobre actitudes en relación a personas con internaciones psiquiátricas prolongadas correspondieron a los ítems de la escala de distancia social de Bogardus ^35^. 

Respecto a la entrevista con comerciantes, esta indagaba por características del barrio, conocimiento de las personas con problemáticas severas de salud mental que residían en el barrio, relación con ellas y observaciones sobre la relación de ellas con los vecinos, y actitudes sobre el tratamiento comunitario y la vida en comunidad de personas com problemáticas severas de salud mental.

### Análisis

El análisis de los cuestionarios fue mixto. Para el análisis cuantitativo se calcularon frecuencias relativas y absolutas, y se analizó la asociación entre algunas variables utilizando chi cuadrado. Respecto al análisis de las preguntas sobre cohesión barrial, se construyó el índice de cohesión barrial, así como los subíndices de sentido de comunidad y de vecindad, considerando que las preguntas tenían una opción de respuesta en escala, asignándose un puntaje de 4 a las respuestas más positivas, y 1 a las más negativas. A partir de ello, y tomando los valores máximos y mínimos que se podían obtener en escala de cohesión barrial y en las subescalas de vecindad y sentido de comunidad, se dividió el puntaje en tres categorías: bajo, medio y alto, y se categorizó a cada participante con una de dichos valores, para cohesión barrial, sentido de comunidad y vecindad. Por su parte, el análisis de las entrevistas con comerciantes fue cualitativo. Se construyó una matriz de análisis tomando como categorías iniciales las preguntas del cuestionario. El material fue analizado por dos investigadores de manera independiente y posteriormente discutido con el equipo ampliado. Respecto a los aspectos éticos, el protocolo fue evaluado por el Comité de Conductas Responsables de la Facultad de Psicología de la Universidad de Buenos Aires, y se utilizó el consentimiento informado con las personas participantes.

## Resultados

### Cuestionario a vecinos y no vecinos

En la Tabla 1 se presentan las características sociodemográficas de las personas a quienes se realizó el cuestionario, comparando vecinos y no vecinos. Como se observa, una característica diferencial entre los vecinos y los no vecinos fue el grupo etario, observándose más adultos mayores en el grupo de vecinos, aspecto que puede explicar otras diferencias sociodemográficas como la ocupación (más amas de casa/jubilados/pensionados en el grupo de vecinos) y la composición del hogar (más hogares unipersonales en el grupo de vecinos).

**Tabla 1:** Caracterización sociodemográfica de vecinos y no vecinos.

Características	Vecinos	No vecinos	Total
	n (%)	n (%)	n (%)
Género			
Femenino	34 (62,0)	42 (66,0)	76 (64,0)
Masculino	20 (36,0)	22 (34,0)	42 (35,0)
Otro	1 (2,0)	-	1 (1,0)
Grupo de edad			
Adulto joven (18-39 años)	12 (22,0)	13 (20,3)	25 (21,0)
Adulto medio (40-64 años)	18 (33,0)	29 (45,3)	47 (39,5)
Adulto mayor (65 años o más)	25 (45,0)	22 (34,4)	47 (39,5)
Media	55 años	53 años	
Mediana	58 años	49 años	
Estado civil			
Soltero	14 (25,5)	15 (23,4)	29 (24,0)
Viudo	12 (22,0)	10 (15,6)	22 (18,0)
Divorciado	3 (5,5)	6 (9,4)	9 (8,0)
Subtotal sin pareja	29 (53,0)	31 (48,4)	60 (50,0)
Casado	21 (38,0)	29 (45,3)	50 (42,0)
Unión de hecho	5 (9,0)	4 (6,2)	9 (8,0)
Subtotal con pareja	26 (47,0)	33 (51,6)	59 (50,0)
Nivel de estudios			
Primaros	9 (16,4)	7 (11,0)	16 (13,0)
Secundarios	20 (36,4)	24 (37,0)	44 (37,0)
Terciarios o universitarios	26 (47,3)	33 (52,0)	59 (50,0)
Ocupación			
Desempleados	4 (7,0)	1 (2,0)	5 (4,2)
Empleados	23 (42,0)	47 (73,0)	70 (58,8)
Ama de casa/Jubilado/Pensionado	28 (51,0)	16 (25,0)	44 (37,0)
Composición del hogar			
Unipersonal	14 (25,5)	11 (17,0)	25 (21,0)
Familiar	38 (69,0)	52 (81,0)	90 (76,0)
Convivencia con no familiares	3 (5,5)	-	3 (2,0)
Sin dato	-	1 (2,0)	1 (1,0)

Respecto a las características psicosociales del vecindario (Tabla 2), en ambos grupos se observa un tiempo prolongado de residencia en el barrio, superior a los 6 años en más del 80% del total de las personas que respondieron al cuestionario. También se observa que más de dos terceras partes de las personas encuestadas tienen un sentido de comunidad alto, que se refiere al sentido de pertenecer o hacer parte del barrio. Por otro lado, el nivel de vecindad reportado permite indicar que las interacciones entre los vecinos en acciones cooperativas se ubican principalmente en los niveles medios y bajos.

**Tabla 2:** Características psicosociales del vecindario.

Características	Vecinos	No vecinos	Total
	n (%)	n (%)	n (%)
Tiempo de residencia en el vecindario			
1 año o menos	1 (2,0)	2 (3,0)	3 (2,5)
2-5 años	6 (11,0)	11 (17,0)	17 (14,3)
6 años o más	47 (85,0)	51 (80,0)	98 (82,3)
Sin dato	1 (2,0)	-	1 (0,8)
Sentido de comunidad			
Bajo	1 (2,0)	3 (4,7)	4 (3,0)
Medio	13 (24,0)	19 (29,7)	32 (27,0)
Alto	41 (74,0)	42 (65,6)	83 (70,0)
Vecindad			
Bajo	18 (33,0)	24 (37,5)	42 (53,0)
Medio	26 (47,0)	31 (48,0)	57 (48,0)
Alto	11 (20,0)	9 (14,0)	20 (17,0)
Cohesión barrial			
Bajo	1 (2,0)	2 (3,0)	2 (2,5)
Medio	33 (60,0)	41 (64,0)	74 (62,2)
Alto	21 (38,0)	21 (33,0)	42 (35,3)

En relación a las actitudes acerca del tratamiento y la vida en comunidad de personas con internaciones psiquiátricas prolongadas, lo primero a señalar es que, pese a que un porcentaje mayor de vecinos que de no vecinos dijo conocer a algún vecino que tuvo una internación psiquiátrica, la diferencia entre ambos grupos no es estadísticamente significativa (χ^2^ = 1,2622; p < 0,05). Tampoco resultan estadísticamente significativas las diferencias entre vecinos y no vecinos sobre las actitudes acerca de la internaciones psiquiátricas prolongadas como modalidad de tratamiento en salud mental: más del 50% de ambos grupos consideró que la internaciones psiquiátricas prolongadas es necesaria, pues hay personas que de ningún modo podrían vivir en la comunidad (χ^2^ = 1,2646; p < 0,05); y también, más de un 50% de ambos grupos consideró que la internaciones psiquiátricas prolongadas termina perjudicando a las personas, más que ayudándolas (χ^2^ = 0,4961; p < 0,05). Es importante señalar que un 30% de la muestra total (n = 36, 17 vecinos, 19 no vecinos) respondió simultáneamente que la internaciones psiquiátricas prolongadas prolongada era necesaria y también perjudicial.

A su vez, entre quienes dijeron conocer a un vecino que tuvo una internación psiquiátrica, un 59% respondió que la internación psiquiátrica era necesaria, y un 65% respondió que la internaciones psiquiátricas prolongadas terminaba perjudicando más a las personas que ayudándolas. Al respecto, no se encontró una diferencia estadísticamente significativa entre quienes dijeron conocer y quienes dijeron no conocer a un vecino que tuvo una internación psiquiátrica y su opinión respecto a la internaciones psiquiátricas prolongadas como necesaria (χ^2^ = 0,0416; p < 0,05), ni tampoco respecto a la internación psiquiátrica prolongada como potencialmente perjudicial (χ^2^ = 0,1853; p < 0,05). Específicamente, dentro del subgrupo de vecinos que dijo conocer a personas del barrio que tuvieron una internación psiquiátrica, un 68% respondió que la internaciones psiquiátricas prolongadas era necesaria, y un 61% respondió que la internaciones psiquiátricas prolongadas podía ser perjudicial.

Por otro lado, en relación al conocimiento y actitudes hacia las personas que tuvieron internaciones psiquiátricas prolongadas (Tabla 3), si se comparan a las personas que dijeron conocer a un vecino que tuvo una internación psiquiátrica con los que dijeron no conocer, y comparar las actitudes de estos dos grupos en relación a la internaciones psiquiátricas prolongadas prolongada como modalidad de tratamiento, los resultados tampoco muestran diferencias estadísticamente significativas. Respecto a considerar a la internaciones psiquiátricas prolongadas como necesaria, un 59% de las personas que dijeron conocer a un vecino que tuvo una internaciones psiquiátricas prolongadas y un 53% de quienes dijeron no conocer, respondieron afirmativamente (χ^2^ = 0,3513; p < 0,05). Por su parte, un 65% de las personas que dijeron conocer a un vecino que tuvo una internación psiquiátrica y un 52% de quienes dijeron no conocer, señalaron que la internaciones psiquiátricas prolongadas podía ser perjudicial (65% de quienes sí conocen y 52% de quienes no conocen, χ^2^ = 1,8939; p < 0,05).

**Tabla 3:** Conocimientos y actitudes en relación al tratamiento y vida en comunidad de personas con internaciones psiquiátricas prolongadas.

	Vecinos	No vecinos	Total
	n (%)	n (%)	n (%)
Conoce vecino que tuvo internaciones psiquiátricas prolongadas	28 (51,0)	26 (41,0)	54 (45,0)
Aceptación del bairro a vecinos com internaciones psiquiátricas prolongadas	26 (47,0)	30 (47,0)	56 (47,0)
Internaciones psiquiátricas prolongadas es necesaria	34 (62,0)	33 (52,0)	67 (56,0)
Internaciones psiquiátricas prolongadas es prejudicial	30 (54,5)	39 (61,0)	69 (58,0)

A su vez, al analizar la relación entre el índice de vecindad y las actitudes hacia la internaciones psiquiátricas prolongadas, no se encontró relación estadísticamente significativa ni en el caso de considerarla necesaria (χ^2^ = 3,3275; p < 0,05), ni en el caso de considerarla perjudicial (χ^2^ = 1,3286; p < 0,05)

**Figura 1:**
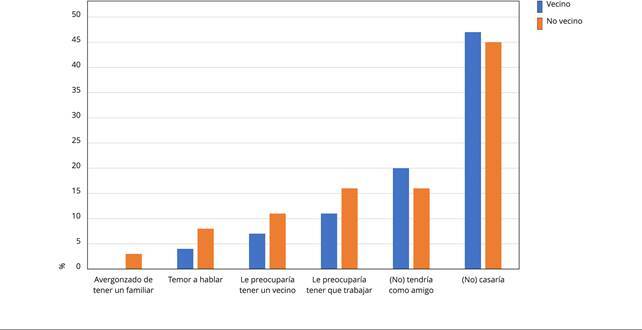
Distancia social hacia personas que estuvieron internadas en un hospital psiquiátrico.

Por otro lado, la Figura 1 muestra la distancia social hacia personas que estuvieron internadas en un hospital psiquiátrico, observándose que esta fue baja en ambos grupos para la mayoría de cuestiones, solo presentando valores de rechazo medio para la pregunta respecto a si se casaría con una persona que estuvo internada en un hospital psiquiátrico.

### Entrevistas a comerciantes de los barrios

Los comerciantes entrevistados trabajaban y vivían en el barrio en su mayoría, y en todos los casos pasaban en el barrio ocho horas o más al día. Se trataba de negocios con un tiempo de funcionamiento en el barrio de más de tres años.

Todas las personas entrevistadas identificaron a los personas con problemáticas severas de salud mental que recibían apoyos por parte de los programas que hacían parte del estudio, y todas indicaron actitudes positivas, aunque con diferentes grados de interacción y cercanía con tales personas. A su vez, y en tanto observadores de la relación entre los vecinos y tales personas, no reportaron problemas, conflictos o rechazo hacia ellas. Una persona entrevistada indicó: “*Hasta ahora, todo tranquilo. Nadie se ha quejado. Un almacén es como un psicólogo que vienen y te cuentan cosas, pero nadie dijo nada*”.

A su vez, se preguntó respecto a las ideas sobre la internaciones psiquiátricas prolongadas: si era considerada como necesaria o bien, si terminaba perjudicando más que ayudando, utilizando las mismas preguntas que en el cuestionario a vecinos y no vecinos. Las respuestas fueron en todos los casos condicionales, es decir, “*dependía del caso*”. Señalaban así que había situaciones en que una persona podía requerir una internación. Una persona señaló: “*Cuando corre riesgo su integridad y la del resto, ahí sí necesitan estar internados*”.

También consideraron que, en base al acompañamiento que observaban que tenían, estas no requerían estar internadas prolongadamente. Es decir, percibían que la vida en la comunidad era posible de sostenerse, en parte gracias al acompañamiento desde los equipos de salud, y que el alta era dada también bajo criterio de los profesionales de salud. Un comerciante comentó: “*Una internación prolongada para estas chicas* ENT#091;se refiere a las personas con problemáticas severas de salud mentalENT#093; *no lo creo. Están apuntaladas, hay gente alrededor. De ahí a si están solas, es otra cosa*”. Otra persona indicó: “*Son gente que está para salir, si no, no las dejarían*”.

## Discusión

Como se señaló anteriormente, no se encontraron diferencias estadísticamente significativas entre vecinos y no vecinos de personas con problemáticas severas de salud mental para ninguna de las variables relacionadas con las actitudes hacia personas con problemáticas severas de salud mental, o el tratamiento en salud mental de tipo comunitario o centrado en la internación prolongada.

Fue baja la distancia social respecto a tener como vecina a una persona que tuvo una internación psiquiátrica, cuando la pregunta se hacía en primera persona, tanto en los vecinos como en los no vecinos (7% y 11% respectivamente). No obstante, el rechazo fue cercano al 50% cuando se preguntaba por la aceptación por parte del barrio al hecho de tener entre los vecinos a alguien que tuvo una internaciones psiquiátricas prolongadas. Esto ha sido descripto como un fenómeno esperable desde la psicología social ^36^, en el sentido de que se suelen atribuir a los demás actitudes más negativas o prejuicios, que a sí mismo. Por otro lado, los estudios previos, tanto en Argentina como en otros países, han arrojado resultados heterogéneos acerca de la distancia social vinculada a tener como vecina a una personas con problemáticas severas de salud mental, con valores medios y bajos ^18^^,^^19^^,^^20^^,^^21^^,^^22^, los cuales pueden deberse a divergencias metodológicas, tanto en los modos de nombrar la variable dependiente, como respecto a la muestra seleccionada. Aun así, cabe destacar que, en comparación con los estudios encontrados, la distancia social identificada en primera persona en nuestro estudio, es la más baja reportada, particularmente entre los vecinos.

Por otro lado, también resulta llamativo que no se observaron diferencias entre personas que dijeron conocer y personas que dijeron no conocer a un vecino que tuvo una internaciones psiquiátricas prolongadas, respecto a su distancia social con personas con problemáticas severas de salud mental, considerando que la teoría e investigación existente indican que el conocimiento y el contacto contribuyen a la reducción del estigma ^37^. Así, resulta de interés continuar profundizando sobre el estudio de las formas de contacto que se producen en contextos de vecindad, y cuáles son los tipos de contacto que contribuyen en tal escenario a la reducción del estigma. En tanto el índice de vecindad identificado en el grupo estudiado fue entre bajo y medio, una posible explicación es que los tipos de contacto son reducidos, esporádicos y no alcanzan a constituirse en insumo para el cambio de actitudes.

El siguiente aspecto a señalar es que no se encontraron diferencias estadísticamente significativas entre vecinos y no vecinos de personas con problemáticas severas de salud mental respecto a sus actitudes en relación a la internación como modalidad de tratamiento en salud mental. Aun así, vale la pena mencionar que el porcentaje de vecinos que consideró a la internaciones psiquiátricas polongadas como necesaria fue mayor que en el caso de los no vecinos, y que, en un sentido opuesto, fue mayor el porcentaje de no vecinos que la consideró como potencialmente perjudicial, en comparación con los vecinos. Posiblemente, las respuestas de los comerciantes de los barrios entrevistados ayuden a comprender este resultado. Según estos, la internación debía ser considerada “*dependiendo del caso*” y en consideración del riesgo para la propia personas con problemáticas severas de salud mental. Es decir, es posible que una persona piense de modo simultáneo que una internaciones psiquiátricas prolongadas es necesaria (dependiendo del caso) y que una internaciones psiquiátricas prolongadas puede terminar perjudicado más que ayudando a una persona (también, dependiendo del caso).

Cabe destacar que las respuestas de los comerciantes respecto a la vida en comunidad de personas con problemáticas severas de salud mental se caracterizaron por descripciones de normalización, y que consideraron que los apoyos a la vida en comunidad brindados por los equipos de salud eran positivos. Así, sería importante continuar analizando las actitudes de quienes conviven comunitariamente con personas con problemáticas severas de salud mental acerca de los apoyos comunitarios en salud mental, a fin de comprender las rupturas y continuidades que ven entre el tratamiento hospitalario y el tratamiento comunitario, desde su experiencia cotidiana. Una línea posible de indagación sería la de analizar las representaciones de la población, dependiendo de la estructura institucional de la cual dependen los servicios de apoyo.

Por otro lado, más del 50% del total de las personas encuestadas respondió que la internaciones psiquiátricas prolongadas es necesaria, pues hay personas que de ningún modo podrían vivir en la comunidad (χ^2^ = 1,2646; p < 0,05); y, también, más de un 50% de ambos grupos consideró que la internaciones psiquiátricas prolongadas termina perjudicando a las personas, más que ayudándolas. Esos valores, comparados con los resultados del estudio de Tostes et al. ^28^ en donde casi el 100% de la muestra analizada no estuvo de acuerdo en que los enfermos mentales debían ser aislados de la comunidad, podrían indicar que la internación prolongada como modalidad de tratamiento aún tiene respaldo entre la población general de Argentina, y que en consecuencia sería pertinente continuar desarrollando materiales comunicacionales que contribuyan a modificar tales creencias.

Vale también destacar que los cuestionarios fueron hechos con una definición muy delimitada de “vecino”: la persona que vivía en las dos viviendas contiguas o de enfrente de las personas que recibían apoyos, y como máxima tolerancia de la misma calle, ello basado en el supuesto de que a mayor cercanía, mayores probabilidades de interacción. Pese a ello, solo la mitad de quienes respondieron al cuestionario, dijeron conocer entre sus vecinos a una persona que tuvo una internación psiquiátrica. Ello puede deberse a varios factores: para empezar, una tercera parte de los vecinos mostró un índice de vecindad bajo, y un 80% entre media y baja, por lo cual podría considerarse que la proximidad y conocimiento de los vecinos fuesen reducidos. A su vez, el que no identifiquen a las personas que reciben apoyos como personas que tuvieron una internación psiquiátrica puede vincularse a que no identifican en ellas cuestiones o conductas que puedan asociarse con haber tenido una internación psiquiátrica.

Finalmente, los resultados de este estudio muestran parte de la experiencia de servicios de apoyo a la vivienda con más de 20 años de desarrollo en Argentina. Por ello, pueden contribuir a que decisores políticos y trabajadores de la salud, contrasten sus discusiones y decisiones, con lo que se observa entre los vecinos de quienes efectivamente comparten el mundo de la vida cotidiana con personas con historia de internaciones psiquiátricas prolongadas. Los resultados aquí presentados indican que no se observa ni aceptación ni rechazo marcado hacia la internación psiquiátrica prolongada, no se observa una distancia social marcada respecto al hecho de ser vecino de alguien que tuvo una internaciones psiquiátricas prolongadas, y a su vez parecieran valorarse los apoyos a la vivienda como un aspecto que ayuda a que la vida en comunidad y la inclusión social sean posibles.
